# Work, malaise, and well-being in Spanish and Latin-American doctors

**DOI:** 10.1590/S1518-8787.2016050005600

**Published:** 2016-04-27

**Authors:** Paola Ochoa, Josep M Blanch

**Affiliations:** I Escuela de Postgrado en Administración de Empresas. Escuela Superior Politécnica del Litoral. Guayaquil, Ecuador; II Departament de Psicologia Social. Facultat de Psicologia. Universitat Autònoma de Barcelona. Barcelona, España

**Keywords:** Physicians, psychology, Working Conditions, Job Satisfaction, Personal Satisfaction, Burnout Professional

## Abstract

**OBJECTIVE:**

To analyze the relations between the meanings of working and the levels of doctors work well-being in the context of their working conditions.

**METHOD:**

The research combined the qualitative methodology of textual analysis and the quantitative one of correspondence factor analysis. A convenience, intentional, and stratified sample composed of 305 Spanish and Latin American doctors completed an extensive questionnaire on the topics of the research.

**RESULTS:**

The general meaning of working for the group located in the quartile of malaise included perceptions of discomfort, frustration, and exhaustion. However, those showing higher levels of well-being, located on the opposite quartile, associated their working experience with good conditions and the development of their professional and personal competences.

**CONCLUSIONS:**

The study provides empirical evidence of the relationship between contextual factors and the meanings of working for participants with higher levels of malaise, and of the importance granted both to intrinsic and extrinsic factors by those who scored highest on well-being.

## RESUMEN

**OBJETIVO:**

Analizar las relaciones entre los significados del trabajo y los niveles de bienestar laboral de los profesionales de la medicina en el contexto de sus condiciones de trabajo.

**MÉTODOS:**

La investigación combinó la metodología cualitativa del análisis textual, y la cuantitativa del análisis factorial de correspondencias. Una muestra de conveniencia, intencional y estratificada, compuesta por 305 profesionales de la medicina de España y de América Latina, respondió un amplio cuestionario sobre los temas de la investigación.

**RESULTADOS:**

El significado general del trabajo del grupo situado en el cuartil de malestar se configuró en torno a percepciones de incomodidad, frustración y agotamiento. En cambio, quienes expresaron mayores niveles de bienestar, ubicados en el cuartil opuesto, asociaron su experiencia laboral a buenas condiciones de trabajo y al desarrollo de las propias competencias profesionales y personales.

**CONCLUSIONES:**

El estudio aporta evidencia empírica acerca de la relación entre factores contextuales y significados de trabajo, en los participantes con mayores niveles de malestar; también, sobre la importancia dada a factores tanto intrínsecos como extrínsecos por quienes obtuvieron mayores puntuaciones en bienestar.

## INTRODUCTION

The current working conditions of the medical sector are characterized by overwork, work pressure, and high levels of psychological work density and intensity^2^ ^,^ ^7^ ^,^ ^14^. The literature shows a variable distribution of psychosocial labor risks by professional categories^11^. The occupational task carried out by medical professionals and health workers in general is characterized by an overload of cognitive and emotional demands and high levels of overtime. This stressful professional activity is performed in a period distinguished by hurry, speed, and urgency. Many medical professionals experience work overload, which refers to an excess of work, sometimes carried out in difficult circumstances, often with a perceived lack of time to finish or to do it properly, and always in a permanent state of being in business and under attendance pressure. Based on this complex combination of circumstances, medical working conditions are considered a potential breeding-ground for burnout^1^ ^,^ ^2^ ^,^ ^7^ ^,^ ^9^ ^,^ ^14^; but also as an opportunity for psychologically positive work experiences^24^ ^,^ ^26^ ^,^ ^28^ ^,^ ^29^ ^,^ ^a^

The complexity and heterogeneity of the countries evaluated in this study, as well as the depth of their contemporary changes, crisis, and social, labor, and economical metamorphosis, require a sociohistorical and multilevel analysis of the labor and professional subjectivity of Latin-American and Spanish doctors. The research focuses on the social, organizational, and psychological effects of new, unstable, and fragmented employment, especially in Latin-American countries^2^, but also in Spain^9^, where the health system fell apart due to a financial and management crisis. Within these processes, a labor and professional crisis also emerges, referring mainly to precariousness caused by job insecurity and work fragmentation, and to work overload and the general working conditions emerging from organizational demands posed by the new management of health centers. This business model for managing these services sometimes drives toward the commercialization of health care as a natural imperative^2^ ^,^ ^8^.

As the labor world is a fundamental scenario of people’s psychological experience, the social and organizational changes in working conditions similarly affect workers’ labor subjectivity, social working relations, professional ethical values and meanings, occupational health, job satisfaction, and work well-being levels.

Research on the meaning of working, work values, and working life is heterogeneous^3^ ^,^ ^8^. Among the numerous empirical studies culminated in the 1980s about what work means for the common people, the outstanding macro report The Meaning of Working (MOW) emerged from a cross-cultural research^23^. The MOW team conceived the meaning of working as a multidimensional construct composed of three main thematic axes: (a) work centrality (importance and value of work as vital role), (b) social norms about working (related to labor role performance), and (c) valued working outcomes and preferred work goals. In the present study, the meaning of working is defined from a sociohistorical perspective as a set of flexible, dynamic, and shared beliefs, attitudes, and values relating to work and professional experience (developed in the historical context of late modern societies). These shared features are established in an identity and assumed, to a greater or lesser degree, by a group of professionals.

The literature about the different types of work well-being is extensive and diverse^5^ ^,^ ^19^ ^,^ ^26^ ^,^ ^28^, showing very close links with topics from Positive Psychology^19^ ^,^ ^a^. Numerous perspectives see well-being as a feeling of satisfaction with life in general and with its relevant aspects, such as working experiences and relations. This kind of experience varies in time and space, as a feeling of life satisfaction and an economic factor^5^ ^,^ ^26^. The objective well-being refers to specific life contexts and the subjective one is related to overall life satisfaction and happiness. Psychological well-being also includes a set of factors concerning human development and existential vital challenges^1^ ^6^. Several studies present the well-being-performance relationship^21^ ^,^ ^30^ and the connection between working conditions and well-being^6^ ^,^ ^9^ ^,^ ^15^ ^,^ ^28^ ^,^ ^29^.

Contemporary work well-being theories are greatly influenced by the ideas of Warr and his ecological model^28^. This psychosociological perspective considers not only workers’ psychological level of satisfaction and fulfillment regarding their working conditions, but also organizational, social, and environmental levels. Work well-being consists of a psychosocial state of cognitive and emotional joyful living, leading to the ability to successfully and flexibly interrelate with job responsibilities and organizational demands. This state depends on the interaction between organizational demands, and personal, social, and cultural resources and competences.

These changes^b^ occur in the context of a profound metamorphosis of the conditions for medical work and of the health care culture driven by old and new forms of public management. Over recent decades, the neoliberal model (less state and more market, less politics and more economics) and the statist paradigm have implemented their “reforms” with varying intensity and effectiveness, and with specific configurations by country.

The Brazilian Federal Constitution from 1988 created the foundations of a Unified Health System (SUS, in Portuguese), which defined health as “everyone’s right and duty of the State”. However, access to health services is not as easy for every citizen, for multiple reasons, as significant social and economic inequalities, disparities between states, deficit of public investment in health infrastructure, public subsidies to the private sector, double coverage of health services for a privileged minority, and the asymmetric distribution of funding SUS between the public and private sectors. All these features greatly affect the quality of the health system and the conditions of medical work^25^ ^,^ ^c^.

The Chilean health system has a long history marked with milestones along the 20^th^ century, among them the creation of the Ministry of Health, Welfare and Social Security in 1924 and of the National Health Service in 1952. The 1981 Reform condensed and summarized the difficult balance between two conflicting tendencies: the movement for the recovery of public health system eroded by the dictatorship and the neoliberal insistence on cutting public spending. At the beginning of this century, new reforms were implemented concerning social rights in health, reorganization of the hospital network, and universal mandatory health plans regulated by the State. In contrast, funding for the system drags large deficits that involve job insecurity, deteriorating working conditions and low quality of work life for the health professionals^4^.

The Colombian health system of the past decades is the heritage of the strengths and weaknesses of Law 100/1993 and its successive reforms of 2007 and 2011. Law 1,752/2015 introduced a new regulation of the fundamental right to health and a new general social security health system. The underlying problems that have been dragging Law 100 were caused by conflictive visions of health as a source of business and as a fundamental right^22^. Despite the fact that health budget has tripled over two decades, access to health services has not become more democratic, and social inequalities in health have increased^11^. These circumstances generated the social perception of deteriorating public health services, working conditions and quality of work life of health professionals.

For its part, the current Spanish health system reflects the tension between hardly compatible objectives and strategies: the goal of excellence in managing the health system^d^ and the severe cuts in their financing imposed by the Ministry of Finance. Moreover, the economic effects of the long crisis that began in 2008, the increase in life expectancy, an aging population, and rising pharmaceutical expenditure, determine the cost of running the health system grow at a rate greater than the economy^29^. In this context, the conditions of medical work in the country have deteriorated by an increase in job instability affecting a growing part of the medical staff, due to the proliferation and extension of their temporary contracts. Furthermore, most medical professionals employed in the public and private sectors have seen their workload increase, in times of budget cuts and staff reductions^e^.

The Venezuelan health system consists of a network of hospitals and clinics financed by the State or by private systems. In the 2000s, the government created a network of health centers and outpatient clinics as part of the *Misión Barrio Adentro*, which includes modules and Comprehensive Diagnostics Centers (CDI). This policy tried to increase the coverage of health services for the poorest populations. However, it failed to focus on fundamental problems of the Venezuelan health system^f^, and the population health conditions have worsened. The current situation^g^ shows high levels of shortages of medicines, reagents, and supplies for diagnostic tests, as well as basic supplies and spare parts for medical equipment for the operation of the health system, both public and private.

The background of the present study consists of the changes in Latin-American and Spanish health systems, the value transformations in health care professionalism, and the growing work overload and job insecurity in flexible jobs within current medical working conditions. In this context, the objective of this research was to analyze the relations between the meanings of working and the levels of doctors work well-being in the framework of their working conditions.

## METHODS

This study combined two approaches: one qualitative, with keywords concerning the meanings of working; and one quantitative, using a self-report survey made up of a battery of scales measuring well-being at work and working conditions.

Pragmatic and epistemological reasons led researchers to seek a compromise between random and convenience sampling. This study combines a long questionnaire, a target-group with little time available to complete it, and a remarkable diversity of contexts among countries regarding opportunities and restrictions in access to random samples. This set of circumstances would determine a very low response rate in random sampling, with consequent problems of statistical representativeness. Following the principles of Grounded Theory^27^, a stratified sampling was used to offset the deficit of statistical representativeness with a reasonable degree of theoretical representativeness. According to this point of view, this quality was ensured by selecting a sufficiently heterogeneous and representative sample of the typological diversity of cases and situations occurring in the medical collective under study.

We conducted a homogeneous collective concerning type of service (health care) and professional sector (medicine) composed of 305 professionals employed in hospitals or other large and complex health centers in Brazil, Chile, Colombia, Venezuela, and Spain. The collective was also somewhat heterogeneous, as all the participants were recruited by convenience, intentional, and stratified sampling, according to six criteria: gender, generation, country, type of contract, seniority in the organization, and whether they had management responsibilities. About half of this group was male, mean age was 42 years, around 40.0% worked in Spain, and the others belonged to various participating Latin-American countries (Brazil, Chile, Colombia, and Venezuela). Seventy-five percent of the participants had stable employment contracts, the juniors and seniors in the organization were equally distributed, and one third had experience in health care services management.

The general tool to collect data was a self-report survey, which also included items concerning sociodemographic information. To analyze the meanings of working, this questionnaire included a free-word association item that invited people to write four keywords defining their current work experience. For the quantitative variables, a standardized battery of scales including the Working Conditions Scale^6^ and the Questionnaire of General Labor Well-being^5^ was used.

The section concerning working conditions included 44 closed items. It was designed according to a theoretical model that configures working conditions around a three-way relationship of the organization with the method, the environment, and the person. A principal component analysis showed the existence of six factors, related to material and social environments, regulation and development methods, organizational adjustment to the person, and adaptation of the person to the organization. The six scales of the theoretical model represented by these factors were rated on an 11-point Likert scale ranging from 0 (very bad, never) to 10 (very good, always).

The Questionnaire of General Labor Well-being included a series of 55 closed items organized according to a theoretical model of general work well-being. Factor analysis yielded two independent factors: the first one, called Psychosocial Well-being, comprised three scales measuring affections, skills, and expectations; and the second one, Collateral Effects, contained three scales evaluating somatization, exhaustion, and alienation. The affections and skills scales had a semantic differential format, ranging from 1 to 7; the expectations scale had a Likert format, ranging from 1 (low) to 7 (high). The three collateral effects scales ranged from 0 (never) to 6 (always).

All scales showed high internal consistency, with Cronbach’s alpha values ranging from 0.80 to 0.97. The psychometric study indicated that the overall instrument reproduced the structure of the proposed theoretical models, and that it was a measurement tool particularly sensitive to the psychosocial dimension of working conditions and of work well-being of health care professionals, and to evaluate the bipolar nature of their well-being experience.

For the qualitative analysis, we used a dual pathway of textual and content analysis, and for the quantitative analysis, we combined descriptive statistics and factorial correspondence analysis, which shows relationships in a set of categorical variables from the contingency table data.

For the textual analysis of lexical forms associated with working experience, we performed thematic analysis of the corpus of data collected sequentially, combining two procedures: bottom-up data classification without adjusting to existing coding frames and top-down recoding, based on a theoretical framework. The result of this process was a dictionary drawn from all the original words of the questionnaire, which was based on a theoretical perspective, facilitating the analysis and discussion of its contents. This theoretical framework was grounded in the burnout and engagement models^14^ ^,^ ^24^ that allows establishing a bipolar axis with burnout-malaise at one extreme and engagement-well-being at the other.

In an initial coding level, general codes related to the malaise and well-being axes were constructed. The categories associated with the malaise pole were negative working conditions, discomfort, exhaustion, cynicism (negative attitude towards the organization), depersonalization (negative treatment of people), and (self-)inefficacy. The categories linked to the well-being pole were positive working conditions, well-being, vigor, commitment, good relationships, personalization, and efficacy.

In a second level of categorization, specific codes were designed including words that condensed groups of meanings from the original terms. This list includes 25 codes, 14 of them with negative connotations: overwork, bad management, disorganization, bad (social) environment, injustice, inappropriate work, lack of resources, instability, dissatisfaction, malaise**,** exhaustion, lack of commitment, depersonalization, and inefficacy. The remaining 11 codes have positive connotations: good (socioeconomic) conditions, opportunities, satisfaction, well-being, empowerment, commitment, ethics, good relations, plenitude, efficacy, and competences. We created an additional category that grouped a low percentage of non-specific responses.

The Ethics Committee of the Autonomous University of Barcelona approved this study. All participants signed an informed consent form. Moreover, in all cases, we applied the international rules on confidentiality of participants and institutions, safeguarding the anonymity of responses, commitment to returning results, and responsible use of information.

## RESULTS

**Figure 1:**
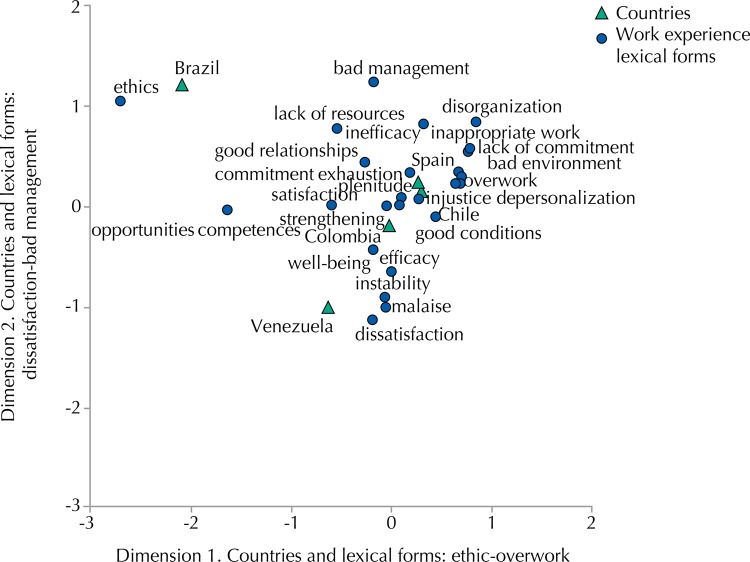
Meaning of working and countries.

All the participants tended to associate medical practice experience in their respective health center to a double type of factors: the valuation of their own working conditions (material, technical, organizational, contractual, temporary, and social); and a set of semantic codes, value systems and beliefs rooted in local cultures of each country, but also very influenced by the respective socioeconomic and health policy joints of every place and time. In this regard, the data obtained show a picture characterized by the following major trends in assessment of working conditions: (a) general convergence between countries on scoring averages ranging between 5.5 and 7.5, depending on factors and scales; (b) no statistically significant differences between the various Latin-American groups; (c) almost statistically significant differences (p = 0.054) between the respective averages of Latin-American and Spanish professionals concerning working conditions in general (tending to show above the first collective); (d) significant differences between Latin-American (mean = 6.89; SD = 1.16) and Spanish (mean = 6.06; SD = 1.28) on the general factor “organization and individuals” (p = 0.001, d Cohen = 0.48). This means that the Latin-American group recognized more adaptation of individual professionals to their organization (accepting the center’s policy, assuming the values of management, identifying themselves with the proposed changes, internalizing the standards, and others). In addition, they perceived a greater degree of accommodation of the organization to the person (regarding satisfaction of individual needs and interests, consistency of culture of the health center with their own personal values, institutional policies affinity with individual aspirations and expectations, and others). The Chilean group showed a special configuration, evaluating their own working conditions close to those of other Latin-American countries scores, but considering their own work experience very close to the Spanish group terms (Figure 1).

The corpus of the meaning of working variable consisted of 161 word forms generated by the participants, with frequencies ranging from 45 to 1 and a total of 769 lexical forms. The distribution quartiles obtained from the responses to the General Labor Well-Being Questionnaire formed the continuous variable well-being. We assigned each response to a category (well-being, normality, risk, and malaise), according to the quartile corresponding to its score on the questionnaire.

A Chi-square test (χ^2^ (75) = 148.820, p < 0.001) led to rejecting the null hypothesis of independence between the meaning of working and the malaise-well-being quartiles and to accepting the alternative hypothesis: the meaning of working and the malaise-well-being quartiles were significantly related.

To determine the nature of this relationship, we performed a Correspondence Analysis. The results showed three factorial axes to explain the total inertia. The first factorial axis had an eigenvalue of 0.440, with a singular factor inertia of 0.193, and total accounted and cumulative inertia of 77.3%. It was the most important axis to explain the relationship between the meaning of work variables and the well-being quartiles. The second factorial axis had a lower eigenvalue, 0.201, with inertia of 0.040, explaining 16.1% of the total inertia. The third axis had an eigenvalue of 0.129, with 0.017 inertia, representing 6.6% of the total inertia. The relevance of the former two factorial axes, which explained 94.1% of the inertia, justified focusing on them, with special emphasis on the first one.

To define the factorial axes from modalities of the variables that contributed to their formation, we described the absolute and relative contributions of the modalities – both the lexical forms of meaning of work experience and the malaise-well-being quartiles – and analyzed the results in the tables and figures. The modalities of the two variables were then projected onto a two-dimensional space.

The first axis (Table) had higher absolute contributions of lexical forms ranging from dissatisfaction (0.110) to good conditions (0.095) and plenitude (0.092). According to its coordinates, at the negative pole of the axis were located lexical forms defining the meaning of working experience as bad management (-1.755), dissatisfaction (-1.668), ineffectiveness (-1.246), depersonalization (-1.045), injustice (-1.018) or instability (-0.870), and at the positive one were situated forms such as good conditions (1.024) and plenitude (0.763).

**Table:** The meaning of working and quartiles of well-being in correspondence analysis.

Variable	Mass	Score in dimension		Inertia	Contribution				

					Of point to inertia of dimension		Of dimension to inertia of point		
		
		1	2		1	2	1	2	Total
Quartiles									
Q1 Malaise	0.255	-0.919	0.442	0.105	0.489	0.248	0.903	0.095	0.999
Q2 Risk	0.244	-0.181	-0.526	0.024	0.018	0.337	0.149	0.571	0.720
Q3 Normality	0.246	0.199	-0.382	0.021	0.022	0.179	0.207	0.349	0.556
Q4 Well-being	0.255	0.901	0.431	0.101	0.470	0.236	0.900	0.094	0.994
Work									
Overwork	0.076	-0.656	-0.580	0.020	0.075	0.128	0.730	0.260	0.990
Bad management	0.009	-1.755	1.240	0.015	0.061	0.066	0.808	0.184	0.992
Disorganization	0.014	-0.616	-1.236	0.007	0.012	0.106	0.319	0.586	0.905
Bad environment (social)	0.017	-0.746	0.484	0.007	0.022	0.020	0.572	0.110	0.682
Injustice	0.019	-1,018	-0.297	0.009	0.045	0.008	0.936	0.036	0.973
Inappropriate work	0.019	-0.042	0.126	0.000	0.000	0.002	0.034	0.141	0.175
Lack of resources	0.017	-0.505	0.335	0.003	0.010	0.010	0.578	0.116	0.694
Instability	0.016	-0.870	-0.237	0.007	0.027	0.004	0.776	0.026	0.802
Dissatisfaction	0.017	-1.668	1.311	0.028	0.110	0.149	0.768	0.216	0.984
Malaise	0.024	-0.832	-0.110	0.008	0.038	0.001	0.880	0.007	0.887
Exhaustion	0.062	-0.780	-0.433	0.019	0.086	0.058	0.877	0.123	10.000
Lack of commitment	0.016	-0.046	-0.329	0.001	0.000	0.008	0.022	0.506	0.528
Depersonalization	0.014	-1.045	1.081	0.010	0.034	0.081	0.665	0.324	0.990
Inefficacy	0.026	-1.246	0.418	0.019	0.092	0.023	0.923	0.047	0.971
Good condition (socioeconomic)	0.040	1.024	0.264	0.019	0.095	0.014	0.969	0.029	0.998
Opportunities	0.019	-0.649	0.505	0.005	0.018	0.024	0.664	0.183	0.848
Satisfaction	0.033	0.763	-0.170	0.009	0.044	0.005	0.943	0.021	0.964
Well-being	0.050	0.411	0.169	0.005	0.019	0.007	0.791	0.061	0.852
Strengthening	0.055	0.460	0.656	0.010	0.027	0.119	0.510	0.474	0.985
Commitment	0.153	0.300	-0.001	0.007	0.031	0.000	0.916	0.000	0.916
Ethics	0.014	0.625	0.040	0.003	0.012	0.000	0.691	0.001	0.692
Good relationships	0.071	0.381	-0.473	0.011	0.023	0.079	0.406	0.286	0.692
Plenitude	0.069	0.763	0.427	0.020	0.092	0.063	0.874	0.125	0.999
Efficacy	0.106	0.308	-0.199	0.006	0.023	0.021	0.732	0.139	0.872
Competencies	0.043	0.183	-0.120	0.001	0.003	0.003	0.777	0.151	0.928

Concerning the absolute and relative contribution of the modalities of malaise-well-being quartiles, the most important axis was defined mainly by the modalities located on the opposite poles malaise (0.489) and well-being (0.470).

The highest relative contributions corresponded to malaise (0.903) and well-being (0.900), which showed that they were well placed in the axis. However, the other modalities of normal and risk were not so well placed. Lexical forms that defined the axis and that were found adequately placed corresponded to the variable meaning of working, but other variables that were also well placed did not contribute to its definition.

To facilitate interpretation of dimension 1, malaise-well-being and their lexical forms, the modalities of the two variables were located on an axis in accordance with their coordinates and absolute and relative contributions. The pole consisting of lexical forms that defined the meaning of working experience for those located in the malaise quartile included bad management, overwork, exhaustion**,** inefficacy, and dissatisfaction. The pole representing the working experience for professionals positioned in well-being quartile included good conditions and plenitude.

The axis risk and malaise and their lexical forms were characterized by the higher absolute contributions of the lexical forms dissatisfaction (0.149), overwork (0.128), strengthening (0.119), disorganization (0.106), and depersonalization (0.081). The examination of its coordinates showed that at the upper end of the axis were located lexical forms that defined the meaning of working experience as dissatisfaction (1.311), bad management (1.240), depersonalization (1.081), and strengthening (0.656), whereas at the lower end of the axis were mainly located disorganization (-1.236) and overwork (-0.580).

Regarding the profile of the malaise-well-being quartiles, as observed in the column profile, the second axis was defined by the modalities risk (-0.337), normality (-0.179) at one extreme, and malaise (0.248) and well-being (0.236) at the other.

At the malaise - well-being pole, the highest relative contribution is strengthening (0.474), and, at the normal-risk pole, disorganization (0.586). These scores indicate that these categories were not well placed on the second axis, and neither was the modality normal.

To view the interpretation of dimension 2, risk-malaise and their lexical forms, the modalities of the two variables were placed on an axis according to their coordinates and absolute and relative contributions. On this axis, one pole includes the lexical forms that define the meaning of working experience as disorganization and overwork for those in the risk-normality quartiles. On the opposite pole, the meaning of working experience in the malaise-well-being quartiles was associated with dissatisfaction, strengthening, and depersonalization.

**Figure 2:**
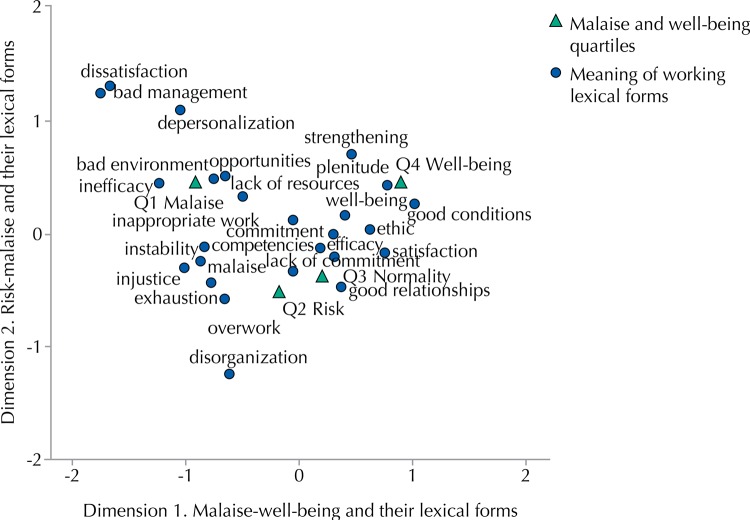
Meaning of working and well-being quartiles.

Figure 2 represents the relation between the meaning of working experience and the malaise-well-being quartiles. The configuration of this meaning for those located in the well-being quartile was made up of the lexical forms of good conditions and satisfaction. For professionals who were in the malaise quartile, working meant inefficacy, dissatisfaction, bad management, overwork, and exhaustion. For individuals in the normality quartile, working meant good condition, and for persons in the risk quartile, it was disorganization.

The meanings of working for the medical group located in the malaise quartile were made up of lexical forms of job discomfort, showing strong evidence of frustration and exhaustion. The set of words included in this category showed that most aspects involve a negative assessment of working conditions and the management of health facilities. Specifically, work experience was described with the terms: bad management (poor plant, lack of perspective, bureaucracy, politicization, and rigidity), work overload (backlog, overwhelming work, hardiness, and sacrifice), exhaustion (burnout, emotional exhaustion, anxiety, and preoccupation), inefficacy (incompetence, worthlessness, and underestimation), and dissatisfaction (disappointment).

In contrast, the job experience of medical professionals who were located in the well-being quartile, including extrinsic and intrinsic aspects, was described as working with good conditions (recognition, compensation, safety, autonomy organization, and flexibility) and plenitude (entertainment, optimal experience, development, enrichment, progress, pride, and self-esteem). Globally, results showed the clear and intense relations between the meanings of working experience and perceived work well-being in the context of specific working conditions.

Figure 1 shows the meanings of working by countries: Brazilian participants meant their work in ethical terms; Venezuelans associated this meaning to dissatisfaction, malaise, and instability, but also to opportunities and responsibilities; Chilean and Spanish professionals, for their part, talked about overload, inappropriate work and inefficacy; and the Colombian meaning of working was positioned on the axes of analysis with difficulties.

## DISCUSSION

This study overviewed how Latin-American and Spanish doctors construct their experiences and meanings of the complex relationship between work, malaise, and well-being in a changing world of health care systems and services. The research provides information about two main points: (a) the close association between specific working conditions and meanings associated with job well-being and professional performance, and (b) the strong link between the meanings that doctors assign to their professional practice and the general environmental characteristics of the settings and contexts where they work. In this regard, situational diversity explains some differences in meaning, whereas shared contextual background tendencies allows better understanding of detected similarities of work, professional values, and ethical dilemmas.

The distribution of keywords on the malaise-well-being axis makes evident the relationship between meaning of working and job well-being experienced by medical professionals. On the malaise quartile, work meanings appear closely associated with worse objective and perceived organizational working conditions (bad management, work overload, exhaustion, bureaucracy, and others). In contrast, scores in the well-being quartile were related to perceived good working conditions and to the importance granted to intrinsic (development, recognition, enrichment, autonomy, and others) and extrinsic (availability of money, security, compensation, and others) factors. The discourses on working and well-being from the surveyed physicians were consistent with data from previous empirical studies^1^ ^,^ ^2^ ^,^ ^7^ ^-^ ^9^ ^,^ ^20^ ^,^ ^31^.

Two single trends in working conditions were reported by most participants as especially problematic common concerns: emerging job insecurity from the temporal instability of job contracts, and the perceived intensification of work due to task overload, which in the health sector was expressed as attendance pressure, the main psychosocial risk factor of professional burnout^1^ ^,^ ^2^ ^,^ ^7^ ^,^ ^9^ ^,^ ^14^ ^,^ ^e^.

The results linking the meanings given to professional experience, job well-being, and working conditions are consistent with the strong association reported between the available material resources of hospitals and the mental health symptoms observed in health care professionals (doctors and nurses)^1^ ^,^ ^9^ ^,^ ^1^ ^4^ ^,^ ^15^ ^,^ ^26^ ^,^ ^28^ ^,^ ^29^ ^,^ ^e^. Furthermore, the findings of this study concerning the negative connotations of the meaning of working for physicians with feelings of work malaise suggest the need to examine in greater depth the cognitive dimension of their experience of work, as burnout studies have suggested^7^ ^,^ ^14^. Likewise, the relationships found between work meanings and the psychosocial dimension of working conditions are consistent with theses about the influence of perceived work environment on one’s evaluation of job experience^2^ ^,^ ^16^. They also follow the same line as Warr’s comments about the impact on working experience, values, and beliefs of the “physical security” dimension, defined as structure, construction, location, safety provisions, ergonomic means, or any other equipment that can provide security against physical threat^28^.

From a sociological and cultural point of view, the data collected suggest an underlying paradoxical process: the historical tension between differentiation and homogenization megatrends. Literature indicates the structural and manifest differences between countries, concerning general economic, social, political, demographic, cultural and historical backgrounds, state and market structures, and health system administration models^2^ ^,^ ^13^ ^,^ ^28^ ^,^ ^29^. The Spanish health system is currently going through a sustainability crisis caused by a political and economic erosion of the European Welfare State system that supports it^e^. The Latin-American cases of Brazil, Chile, Colombia, and Venezuela reflect very different contemporary ways of coping with the challenges of their respective health systems^1^ ^,^ ^2^ ^,^ ^4^ ^,^ ^10^ ^,^ ^12^ ^,^ ^20^ ^,^ ^21^ ^,^ ^b^. In contrast with those centrifugal trends towards differentiation between countries, emerge signs of a centripetal tendency towards assimilation between them, caused by the shared historical, global tendencies towards redesigning work and organization and towards the reformation of the model of state and public services^2^ ^,^ ^e^.

The joint impact of these conflicting processes is visible in the meanings given by doctors to their professional practice, closely related to their perception of the quality of their own specific organizational environment. Spanish and Chilean participants shared their respective working experiences, commonly described as an overloaded and monotonous agenda, and bureaucratic, politicized, inflexible, and ineffective management. These professionals considered work overload as the worst of their working conditions and as a main psychosocial risk factor of burnout. Another perceived major cause of professional discontent in the daily routine was job insecurity. Spanish participants with unstable jobs (temporary contract) reported lack of career prospects in a public service system^e^. Likewise, Chilean doctors reported a similar experience in a privatized system. The present Spanish context of persistent general crisis, affecting the dynamics of the health system, generates a relatively pessimistic vision of the medical work and profession in the participants. In the Chilean case, chronic problems concerning health services were seen as dealt with by reforms^4^.

The professional practice of Colombian doctors also showed their discomfort with temporary contracts, low pay, difficulties of the public health care system, and frustration for failing to provide good service to the community. Thus, they conveyed a critical point of view about the negative working conditions of medical care, affected by some aspects of the reforms carried out during the past few years. They also expressed a shared belief in the need to seek greater coverage and equity in health services for most people.

The meanings given by Venezuelan doctors to their work reflected a difficult experience of tension between vocation requirements and the limited resources to carry out their profession, strongly influenced by the progressive deterioration of the national public health system in recent decades, characterized by lack of supplies for diagnostic tests, lack of medicines, infrastructure problems, and precarious working conditions.

Over the last few years, the Brazilian government invested in health personnel and infrastructures, developing public health policies. In this context, participants of this country showed a confuse representation of work, which some describe as an uncomfortable experience and others as an opportunity to put professional and ethical principles into practice^1^ ^,^ ^20^ ^,^ ^21^.

Moreover, within this basic diversity, the various countries also share a more or less successful history of health system reforms. Despite their remarkable variety of achievements and strategies, they were oriented toward the similar main goal of increasing the health system efficiency and redesigning the management and direction of the public health system, health care centers, services, and working conditions of medical professionals. This historical innovation has generated new organizational subcultures and professional practices facing a challenging, difficult, and conflicting cultural combination of values, such as efficiency and empathy, cost-benefit analysis and the Hippocratic path, business and humanism, common good and private benefit, commercial and social demands, managerial and professional priorities, market laws and the personal quality of working life^7^ ^-^ ^9^ ^,^ ^31^.

This underlying historical background and cultural matrix is an important explanatory key to the similar meanings constructed by professionals living and working in such heterogeneous settings, crossed by conflicting megatrends. They described their experience of reforms as changes, bureaucratization, politization, or reorganization, and noted certain dominant values of the new model of managing health system, such as flexibility, efficiency, competitiveness, demand-resources, financing, profit, client, quality, or cost-benefit. The shared appraisal of the health system reforms by most respondents to the questionnaire provides evidence of an unbalanced process: perceived positive general advances concerning infrastructural and organizational investments and negative outcomes from the new management of an increasing number of patients and health care professionals.

In this way, despite their contextual diversity, doctors from different countries tended to acknowledge certain good organizational, material, and technical working conditions, which allow them to work more and better with patients. However, they expressed also discomfort about work overload, time pressure, and certain ethical and professional strains and dilemmas posed by new organizational demands, economical restrictions, and working conditions. They also criticized reform strategies and practices for being insufficient to promote health and well-being policies for medical and other health care professionals. Thus, they showed the discrepancy between the official rhetoric about the need to develop human talent as a strategic resource in health care organizations (because of its impact on productivity and quality of job performance) and the practical inattention of the psychosocial dimension of medical professional risks and their quality of working life^1^ ^,^ ^2^ ^,^ ^10^ ^,^ ^20^ ^-^ ^22^ ^,^ ^31^ ^,^ ^b^.

Another link among the common cultural background of the surveyed workers was the manifest centrality of work in their personal and social life and of values of medical professionalism (responsibility, involvement, ethics, vocation, service, and others) in the entire sample of participants in the study. Participants showed it independently of their present political context, working conditions, relative job satisfaction, or specific organizational circumstances of their professional practice.

Our data are consistent with the information provided by the literature^,^ ^1^ ^,^ ^2^ ^,^ ^7^ ^-^ ^9^ ^,^ ^12^ ^,^ ^13^ ^,^ ^19^ ^,^ ^29^ ^,^ ^31^ ^,^ ^e^. They also illustrate the benefits that the World Health Organization^20^ and the American Psychological Association^a^ called healthy workplaces for professional health and job performance of health care workers. They indicated the need to promote the pleasant aspect of working conditions in the investigated contexts in order to generate work well-being. And also to prevent the negative effects of the dark side of working conditions, such as risk factors of job malaise and discontent in professionals and low rates of productivity and efficiency in their service to the community^1^ ^,^ ^2^ ^,^ ^14^ ^,^ ^16^ ^,^ ^2^ ^6^ ^,^ ^28^.

The present research has limitations and strengths. The adopted theoretical sampling procedure does not allow statistical generalization of the obtained data to the reference population. However, this *stricto sensu* non-epidemiological research enhances our theoretical understanding of current relevant emerging professional experiences in this population. Moreover, the study performed a combination of qualitative and quantitative methodological strategies, using a design of text dictionary and adopting the bipolar malaise-well-being axis. The empirical work promoted the participation of professionals from five countries, focusing on a profession that involves important psychosocial risks. The results point out some aspects to consider in the preventive political agenda aimed at avoiding, eliminating, or minimizing risk factors as well as promoting healthy organizational conditions of the professional medical practice.

## Footnotes

a American Psychological Association. Psychologically healthy workplace awards. Washington (DC); 2012. Available from: http://www.apaexcellence.org/

b Comisión Económica para América Latina y el Caribe. El panorama social de América Latina. Santiago de Chile: CEPAL; 2013. Available from: www.cepal.org/es/publicaciones/panorama-social-de-america-latina-2013

c Instituto Brasileiro de Geografia e Estatística. Sintese de indicadores sociais: uma análise das condiçoes de vida da população brasileira. Rio de Janeiro; 2010 [cited 2015 Oct 9]. Available from: http://www.ibge.gov.br/home/estatistica/populacao/condicaodevida/indicadoresminimos/sinteseindicsociais2010/SIS_2010.pdf

d Ministerio de Sanidad, Servicios Sociales e Igualdad. Informe anual del Sistema Nacional de Salud. Madrid; 2013 [cited 2015 Oct 9]. Available from: http://www.msssi.gob.es

e Organización Médica Colegial de España. Estudio sobre la situación laboral de los médicos en España. *Acta Sanit*. 2014 [cited 2014 Feb 2]. Available from: http://www.actasanitaria.com/wp-content/uploads/2014/11/medicos-laboral.pdf

f D’Elia Y, Quiroz, C. Las misiones sociales: ¿una alternativa para superar la pobreza? Caracas: Instituto Latinoamericano de Investigaciones; 2010 [cited 2015 Oct 9]. Available from: http://www.ildis.org.ve/website/administrador/uploads/uploads/DocumentoYolanda MisionesPolSocial.pdf

g Provea. Crítica situación del derecho a la salud en Venezuela. *Bol Int Derechos Human* [Internet]. 2014 Jul-Ago [cited 2015 Oct 9];(13). Available from: http://www.derechos.org.ve/2014/09/12/critica-situacion-del-derecho-a-la-salud-en-venezuela

## References

[1] Ansoleaga Moreno E, Toro JP, Godoy L, Stecher A, Blanch JM (2011). Malestar psicofisiológico en profesionales de la salud pública de la Región Metropolitana. Rev Med Chile.

[2] Ansoleaga Moreno E, Artaza Barrios O, Suárez Jiménez JM (2012). Personas que cuidan personas: dimensión humana y trabajo en salud.

[3] Ardichvili A, Kuchinke KP (2009). International perspectives on the meanings of work and working: current research and theory. Adv Dev Hum Resour.

[4] Artaza O, Ansoleaga Moreno E, Artaza Barrios O, Suárez Jiménez JM (2012). La reforma a la salud en Chile. Personas que cuidan personas: dimensión humana y trabajo en salud.

[5] Blanch JM, Sahagún M, Cantera L, Cervantes G (2010). Cuestionario de bienestar laboral general: estructura y propriedades psicométricas. Rev Psicol Trab Organ.

[6] Blanch JM, Sahagún M, Cervantes G (2010). Estructura factorial del cuestionario de condiciones de trabajo. Rev Psicol Trab Organ.

[7] Blanch JM, Crespo FJ, Sahagún MA, Ansoleaga Moreno E, Artaza Barrios O, Suárez Jiménez JM (2012). Sobrecarga de trabajo, tiempo asistencial y bienestar psicosocial en la medicina mercantilizada. Personas que cuidan personas: dimensión humana y trabajo en salud.

[8] Blanch JM, Ochoa P, Sahagún MA, Ansoleaga Moreno E, Artaza Barrios O, Suárez Jiménez JM (2012). Resignificación del trabajo y de la profesión médica bajo la nueva gestión sanitaria. Personas que cuidan personas: dimensión humana y trabajo en salud.

[9] Blanch JM (2014). Quality of working life in commoditized hospitals and universities. Papel Psicol.

[10] Sociales Comisión para Reducir las Desigualdades, Salud n, España n (2012). Propuesta de políticas e intervenciones para reducir las desigualdades sociales en salud en España. Gac Sanit.

[11] Flórez  CE, Camacho A (2012). Dos décadas de cambios en la equidad del sistema de salud colombiano: 1990-2010.

[12] Frenz P, Titelman D (2013). Equidad en salud en la región más desigual del mundo: un reto de políticas públicas en America Latina. Rev Peru Med Exp Salud Publica.

[13] García-Ramírez J, Vélez-Álvarez C (2013). América Latina frente a los determinantes sociales de la salud: políticas públicas implementadas. Rev Salud Publica.

[14] Gil-Monte PR, Moreno-Jiménez B (2007). El síndrome de quemarse por el trabajo (burnout): grupos profesionales de riesgo.

[15] Grebner S, Semmer NK, Elfering A (2005). Working conditions and three types of well-being: a longitudinal study with self-report and rating data. J Occup Health Psychol.

[16] Haller M, Hadler M (2006). How social relations and structures can produce happiness and unhappiness: an international comparative analysis. Soc Indic Res.

[17] Moreno Moreno MC, López López MV (2009). La salud como derecho en Colombia 1999-2007. Rev Gerenc Polit Salud.

[18] MOW International Research Team (1987). The meaning of working.

[19] Noblet A, Cooper C, McWilliams J, Rudd A (2007). Well being, job satisfaction and commitment among Australian community health workers: the relationship with working conditions. Aust J Prim Health.

[20] Organización Mundial de la Salud, Organización Panamericana de la Salud (2010). Ambientes de trabajo saludables: un modelo para la acción: para empleadores, trabajadores, autoridade normativas y profesionales.

[21] Organización Panamericana de la Salud (2005). Análisis del sector salud: una herramienta para viabilizar la formulación de políticas: lineamientos metodológicos.

[22] Organización Panamericana de la Salud (2007). Capacidades en salud pública en América Latina y el Caribe: evaluación y fortalecimiento.

[23] Peiró M, Barrubés J (2012). Nuevo contexto y viejos retos en el sistema sanitario. Rev Esp Cardiol.

[24] Salanova M, Schaufeli WB (2009). El engagement en el trabajo: cuando el trabajo se convierte en pasión.

[25] Santos IS, Uga MAD, Porto SM (2008). O mix público-privado no Sistema de Saúde Brasileiro: financiamento, oferta e utilização de serviços de saúde. Cienc Saude Coletiva.

[26] Sousa-Poza A, Sousa-Poza AA (2000). Well-being at work: a cross-national analysis of the levels and determinants of job satisfaction. J Soc Econ.

[27] Strauss AL, Corbin JM (1990). Basics of qualitative research: grounded theory procedures and techniques.

[28] Warr PB (2007). Work, happiness and unhappiness.

[29] Wilson MG, Dejoy DM, Vandenberg RJ, Richardson HA, Mcgrath AL (2004). Work characteristics and employee health and well-being: test of a model of healthy work organization. J Occup Organ Psychol.

[30] Wright TA, Cropanzano R, Bonett DG (2007). The moderating role of employee positive well being on the relation between job satisfaction and job performance. J Occup Health Psychol.

[31] Wynia MK (2008). The short history and tenuous future of medical professionalism: the erosion of medicine’s social contract. Perspect Biol Med.

